# Autophagy modulates the metabolism and growth of tomato fruit during development

**DOI:** 10.1093/hr/uhac129

**Published:** 2022-06-13

**Authors:** Saleh Alseekh, Feng Zhu, José G Vallarino, Ewelina M Sokolowska, Takuya Yoshida, Susan Bergmann, Regina Wendenburg, Antje Bolze, Aleksandra Skirycz, Tamar Avin-Wittenberg, Alisdair R Fernie

**Affiliations:** Max-Planck-Institute of Molecular Plant Physiology, 14476 Potsdam-Golm, Germany; Center of Plant Systems Biology and Biotechnology, 4000 Plovdiv, Bulgaria; Max-Planck-Institute of Molecular Plant Physiology, 14476 Potsdam-Golm, Germany; National R&D Center for Citrus Preservation, Key Laboratory of Horticultural Plant Biology, Ministry of Education, Huazhong Agricultural University, 430070 Wuhan, China; Max-Planck-Institute of Molecular Plant Physiology, 14476 Potsdam-Golm, Germany; Max-Planck-Institute of Molecular Plant Physiology, 14476 Potsdam-Golm, Germany; Max-Planck-Institute of Molecular Plant Physiology, 14476 Potsdam-Golm, Germany; Max-Planck-Institute of Molecular Plant Physiology, 14476 Potsdam-Golm, Germany; Max-Planck-Institute of Molecular Plant Physiology, 14476 Potsdam-Golm, Germany; Max-Planck-Institute of Molecular Plant Physiology, 14476 Potsdam-Golm, Germany; Max-Planck-Institute of Molecular Plant Physiology, 14476 Potsdam-Golm, Germany; Boyce Thompson Institute, 14850, Ithaca, US; Max-Planck-Institute of Molecular Plant Physiology, 14476 Potsdam-Golm, Germany; Current Address: Department of Plant and Environmental Sciences, Alexander Silberman Institute of Life Sciences, The Hebrew University of Jerusalem, Givat Ram, Jerusalem, 9190401; Max-Planck-Institute of Molecular Plant Physiology, 14476 Potsdam-Golm, Germany; Center of Plant Systems Biology and Biotechnology, 4000 Plovdiv, Bulgaria

## Abstract

Although autophagy is a conserved mechanism operating across eukaryotes, its effects on crops and especially their metabolism has received relatively little attention. Indeed, whilst a few recent studies have used systems biology tools to look at the consequences of lack of autophagy in maize these focused on leaf tissues rather than the kernels. Here we utilized RNA interference (RNAi) to generate tomato plants that were deficient in the autophagy-regulating protease *ATG4*. Plants displayed an early senescence phenotype yet relatively mild changes in the foliar metabolome and were characterized by a reduced fruit yield phenotype. Metabolite profiling indicated that metabolites of *ATG4*-RNAi tomato leaves just exhibited minor alterations while that of fruit displayed bigger difference compared to the WT. In detail, many primary metabolites exhibited decreases in the *ATG4*-RNAi lines, such as proline, tryptophan and phenylalanine, while the representative secondary metabolites (quinic acid and 3*-trans*-caffeoylquinic acid) were present at substantially higher levels in *ATG4*-RNAi green fruits than in WT. Moreover, transcriptome analysis indicated that the most prominent differences were in the significant upregulation of organelle degradation genes involved in the proteasome or chloroplast vesiculation pathways, which was further confirmed by the reduced levels of chloroplastic proteins in the proteomics data. Furthermore, integration analysis of the metabolome, transcriptome and proteome data indicated that ATG4 significantly affected the lipid metabolism, chlorophyll binding proteins and chloroplast biosynthesis. These data collectively lead us to propose a more sophisticated model to explain the cellular co-ordination of the process of autophagy.

## Introduction

In the lytic organelle, autophagy is a conserved eukaryotic mechanism for the breakdown of cellular components (vacuole in yeast and plants and lysosome in animals) [1, 2]. The targets of autophagy are varied and range from single proteins to protein complex and whole organelles [3]. Plants contain three forms of autophagy: macroautophagy, microautophagy, and megaautophagy; macroautophagy is the most well-studied (hereinafter referred to as “autophagy”) [4]. Autophagy starts with the appearance of phagophores, which are cup-shaped double-membranes in the cytoplasm that absorb cytoplasmic particles. The double-membrane then closes to produce double-membrane vesicles, autophagosomes, which travel to the vacuole. The outer membrane of the autophagosome fuses with the tonoplast to release a single-membrane autophagic body in the vacuolar lumen. The autophagic body and its content are degraded in the vacuole, and the resulting building blocks are exported back to the cytoplasm for reuse [4].

The molecular mechanism of autophagy is mediated by autophagy-related (ATG) genes, participating in autophagy induction, autophagosome formation and targeting to the vacuole. The genes are extremely conserved, and *ATG* homologs have been found in a variety of plant species [5]. Most of the research regarding autophagy in plants has focused on the model plant Arabidopsis (*Arabidopsis thaliana*). However, since its characterization in Arabidopsis, autophagy has been seen in a variety of plant species, including the model algae Chlamydomonas (*Chlamydomonas reinhardtii*) and various crop plants such as tomato (*Solanum lycopersicum*), wheat (*Triticum aestivum*), maize (*Zea mays*) and potato (*Solanum tuberosum*) [6–9].

The suite of autophagy-deficient (*atg* mutant) plants are shown to be hypersensitive to nutrient starvation, display decreased yield and early senescence [10]. Interestingly, recent work in which *ATG* genes has been overexpressed in Arabidopsis exhibited increased yield [11], further emphasizing autophagy’s significance in yield determination. Furthermore, autophagy has been proven to play an important function in plant abiotic stress management [3, 12] as well as function in plant-pathogen interactions [13]. In correspondence with autophagy’s role as a recycling mechanism, the metabolic content has been found to be dramatically changed in the *atg* mutants under both favourable and starvation conditions [14, 15]. These results implicate autophagy as a regulator of cellular metabolism. Although several researches have reported that autophagy can significantly change the lipid, primary and secondary metabolism in rice and Arabidopsis [16, 17], the information about autophagy’s role in tomato cellular metabolism remains limited.

Regarding its role in yield determination, autophagy has been shown to be involved in nitrogen remobilization from the mother plant to the seeds [10, 18], as well as function during seed development [19]. However, thus far, the only plant species examined were Arabidopsis and maize, which produce mostly dry seeds and do not possess a fleshy fruit. Since fruit development is accompanied by many metabolic changes [20, 21], and the role of senescence in the process has recently been established [22], we were interested in examining the contribution of autophagy to fruit development, focusing on the pericarp. In the current work, we generated tomato plants expressing an RNA interference (RNAi) construct against the tomato *ATG4* gene. Plants with decreased *ATG4* expression (*ATG4*-RNAi plants) displayed an early senescence and a lower yield phenotype, as well as altered transcript and particularly protein and metabolite content during fruit development. To our knowledge, this is the first report about the involvement of autophagy in the fruit pericarp metabolic content.

## Results

### Tomato plants with reduced *ATG4* expression display early senescence and reduced yield

**Figure 1:**
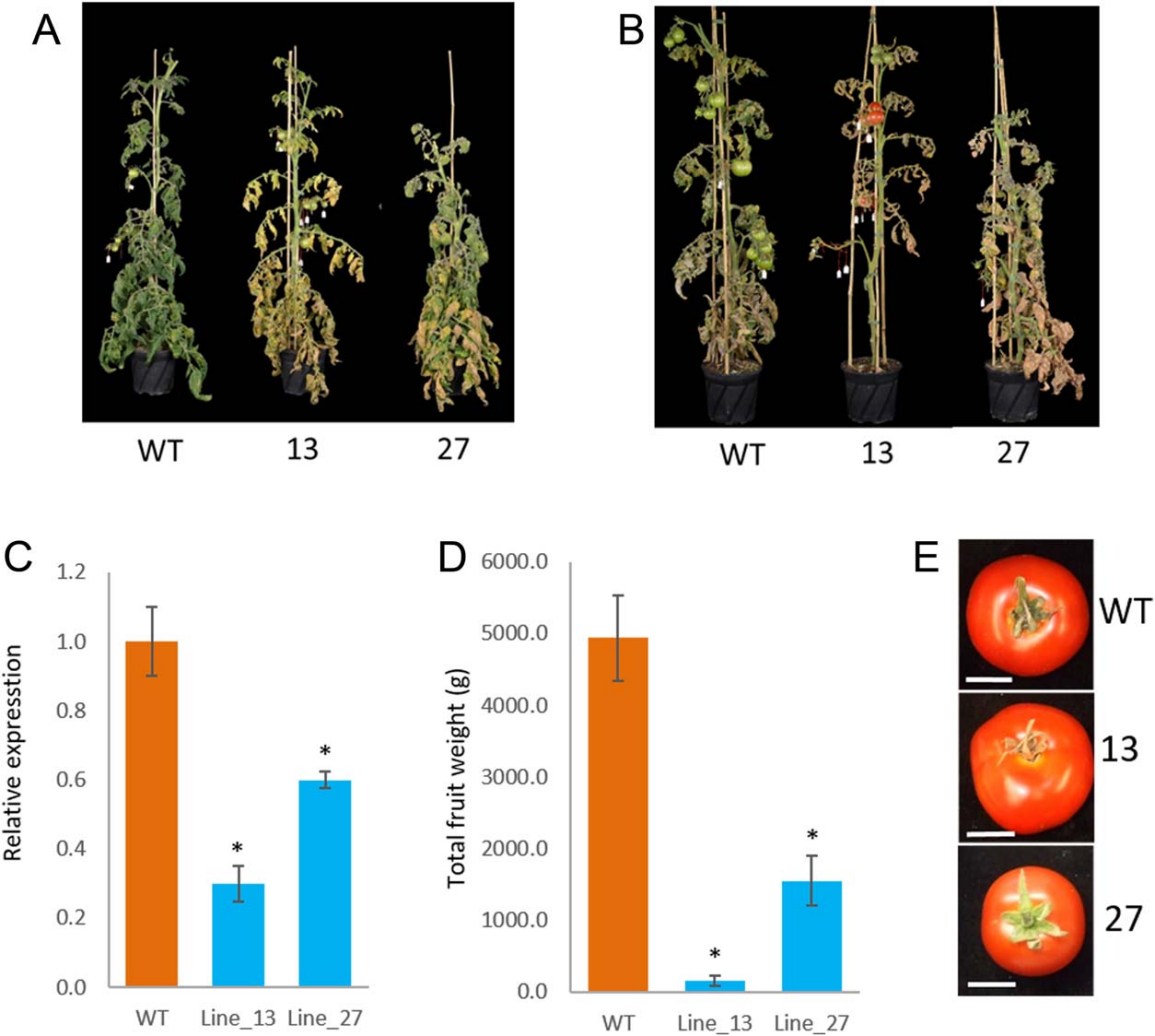
**ATG4 RNAi plants display induce senescence and reduce fruit yield in comparison to wild-type plants.** (A) and (B) representative images of WT and *ATG4*-RNAi plants at the mature green stage and red fruit stage, respectively. Line 13 – presenting with the most severe phenotype, plants have been transferred to the greenhouse at the same time. (C) relative expression of *ATG4* in leaf samples of WT and *ATG4*-RNAi transgenic plants was evaluated by qRT-PCR. (D) total fruit weight of WT and *ATG4*-RNAi plants. (E) Representative image of red fruit from WT and *ATG4*-RNAi lines, scale: 2 cm. Data is presented as average ± SE. Asterisks denote *p* < 0.05 compared to WT in a Student’s *t*-test (*n* = 3–4).

In order to examine the role of autophagy in tomato plants, we generated transgenic tomato expressing an RNAi construct designed to target the tomato *ATG4* gene (Solyc01g006230). *ATG4* is a single gene in tomato, as opposed to two homologs in Arabidopsis [23]. Transgenic tomato plants were propagated by cuttings, and leaf samples were collected for qRT-PCR analysis of *ATG4* expression. Based on the genotyping by NPT-II specific primer and qRT-PCR analysis of *ATG4* expression of nine lines, two lines displayed significantly reduced gene expression (line 13 and line 27) and were selected for further analysis and all the following experiments were used the T3 generation materials. The plants were transferred to the greenhouse and grown until fruit set and maturation. The *ATG4*-RNAi lines displayed a clear early senescence phenotype compared to the control lines (WT). The phenotype was visible from the appearance of green fruit onwards (Figure 1A) and was exacerbated as the plants aged (Figure 1B). The most severe phenotype was observed for *ATG4*-RNAi-13, correlating with the lowest expression of *ATG4* (Figure 1C).

As one of the hallmark phenotypes of autophagy-defective mutants (*atg* mutant) is early senescence [24], we examined whether the *ATG4*-RNAi lines displayed another *atg* mutant phenotype, namely reduced yield. T3 generation of *ATG4*-RNAi lines along the WT grow in the green house, each with three or four replicates, we counted all the fruit for each replicate, up to six fruits per replicate were harvested, and measure their weight, total weight was calculated by multiplying the average of harvested fruits to number of fruits per line. This revealed that the *ATG4*-RNAi lines exhibited a significant reduction in total fruit weight compared to the control lines (Figure 1D, E). The total fruit weight correlated with *ATG4* expression, with *ATG4*-RNAi-13 demonstrating the lowest total fruit weight, and *ATG4*-RNAi-27 showing an intermediate total fruit weight. Moreover, because the early senescence of *ATG4*-RNAi lines (Figure 1A, B) may affect the growth of plant and the development of floral meristems and fruits, the reduced yield may be an indirect result of early senescence.

### *ATG4*-RNAi tomato leaves display only minor alterations in metabolite levels

**Figure 2:**
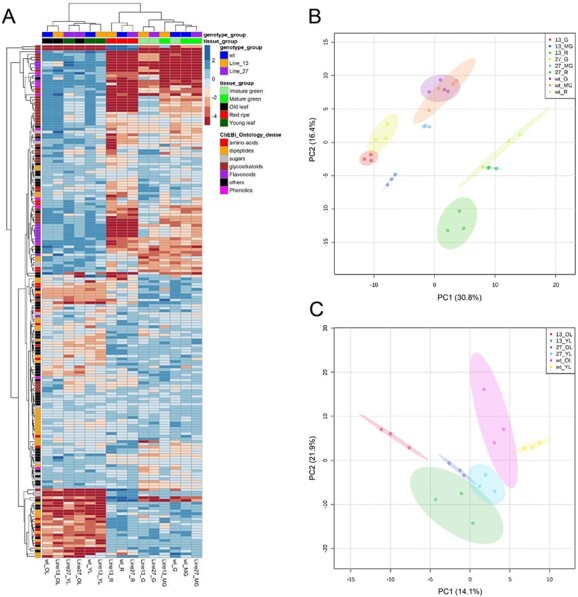
**Metabolic changes between WT and *ATG4*-RNAi plants during fruit development.** WT and *ATG4*-RNAi were grown in the greenhouse. (A) heat map of pericarp tomato fruit at three developmental stages (green, mature green and red) and young and old leafs were collected and analyzed by GC–MS and LC–MS (*n* = 4–6). (B) a PCA of fruit metabolite profiling of WT and *ATG4*-RNAi plants from three developmental stages (G: immature, MG: mature green and R:red ripe). (C) a PCA of metabolite profiling from young leaf (YL) and old leaf (OL) of WT and *ATG4*-RNAi plants. A heat map depicts the log2 values of relative metabolite levels. The levels of *ATG4*-RNAi were normalized to the average of all samples. The metabolites are divided into amino acids, dipeptides, sugars, glycoalkloids, flavonoids, and phenolics.

To examine the effect of *ATG4* downregulation on the leaf metabolites, we collected young (developing not full expanded) and old leaf (full expanded) and analysed their primary, secondary metabolite content and dipeptide abundance by gas chromatography–mass spectrometry (GC–MS) and liquid chromatography–mass spectrometry (LC–MS)-based protocols. The heat map and principle component analysis (PCA) of the leaf and fruit metabolites indicated that the samples of each genotype were largely grouped together and there were only slightly separated, which reveals the small difference between the *ATG4*-RNAi lines and the control lines (Figure 2A-C). In detail, several primary metabolites, such as citric acid in young leaf and glycerate, GABA, and dehydroascorbate in the old leaf, were significantly altered in both lines 13 and 27 compared to WT, respectively. Moreover, other primary metabolites (such as rhamnose, fumarate, malate, and *myo*-inositol in young leaf and phenylalanine in the old leaf also decrease but exhibited significant differences in only a single *ATG4*-RNAi line (Supplementary Data Set S1).

Given that loss of autophagy has previously been reported to result in alterations in secondary metabolite and dipeptide content [15], the metabolite profiling based on LC–MS annotated 224 metabolites, including flavonoids, glycoalkaloids, phenolics, and dipeptides and indicated that impairment of autophagy resulted in significant decrease effect on leaf secondary metabolites and dipeptides. In young leaves, 3,5-dimethoxy-4-hydroxycinnamic acid, alpha-aminopimelic acid, alpha-Ketoglutaric acid and pipecolinic acid were significantly decreased in both *ATG4*-RNAi lines and in old leaf, pyridoxic acid, panthenol while quercetin-*3-O-6-O*-(rhamnosyl) and glucoside-*7-O*-rhamnoside were significantly decreased and increased in both two *ATG4*-RNAi lines (Supplementary Data Set S1), respectively. Moreover, based on our reference library, we annotated 44 dipeptides. However, the effect of autophagy on leaf dipeptides was varied, and no dipeptides exhibited a stable difference in both of *ATG4*-RNAi lines in young and old leaves.

### Tomato *ATG4*-RNAi fruit display altered metabolic content as they ripen

**Figure 3:**
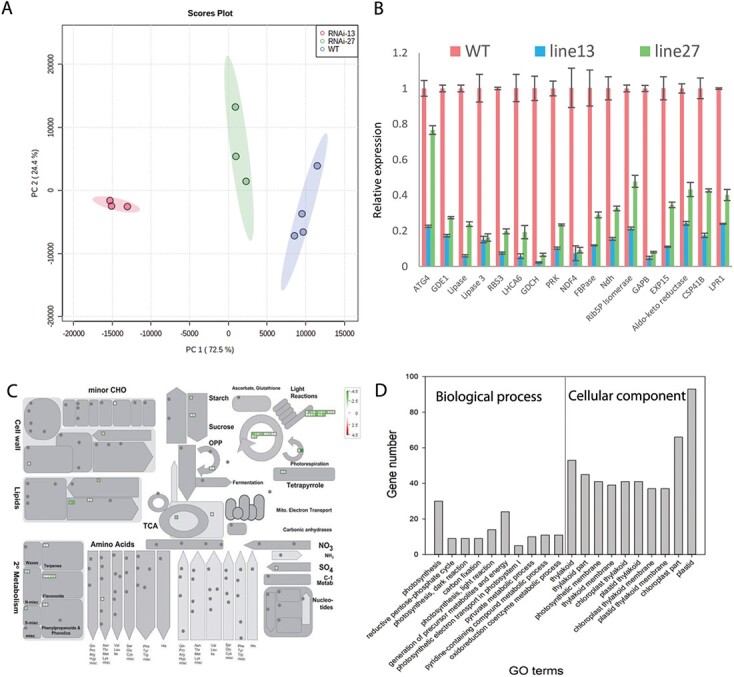
**Differential transcriptome analysis between WT and *ATG4*-RNAi plants at green mature fruit.** (A) Principal component analysis (PCA). (B) The difference of representative genes expression between WT and *ATG4*-RNAi lines. Data is presented as average ± SE. (C) Schematic of major pathways and processes using the MapMan visualization platform of the differentially expressed genes. (D) Gene Ontology enrichment analysis results for differentially expressed genes, The Y axis represents the number of differentially expressed genes; the X axis represents the Gene Ontology functional classification.

**Figure 4:**
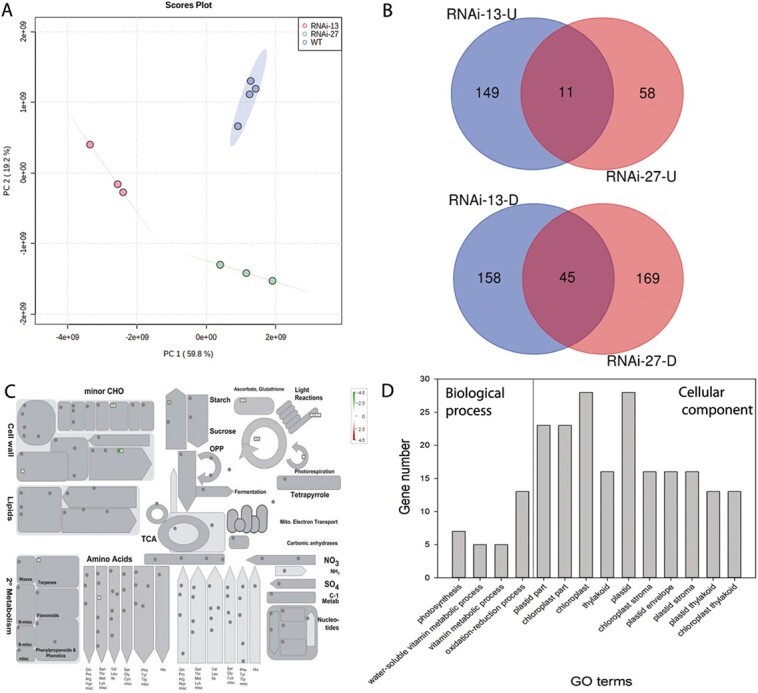
**Differential proteomes analysis between WT and *ATG4*-RNAi plants at green mature fruit.** (A) Principal component analysis (PCA) and (B) Venn diagrams of differential abundant proteins measured by LC–MS analysis. The Venn diagram showing the upregulated (-U) and downregulated (-D) proteins in *ATG4*-RNAi plants compared to the WT. (C) Schematic of major pathways and processes using the MapMan visualization platform of the differentially abundant proteins. (D) Gene Ontology enrichment analysis results for differentially abundant proteins, The Y axis represents the number of differentially expressed proteins; the X axis represents the Gene Ontology functional classification.

To further examine the effect of *ATG4* downregulation on the fruit pericarp, we collected fruit samples at imature green (17 days post-anthesis, DPA), mature green (39 DPA) and red fruit stages (52 DPA) for the metabolite profiling. Principle component analysis (PCA) of the metabolites revealed separation of the samples along PC1, corresponding to fruit ripening (Figure 2B). These results are not surprising seeing that tomato fruit undergo distinct metabolic shifts throughout fruit ripening [20]. When we compared the metabolic profiles of *ATG4*-RNAi lines to control lines in different fruit development stages, we could see that, indeed, both *ATG4*-RNAi lines were grouped together and significantly separated with WT sample at the green fruit stage and during the mature green and red fruits, *ATG4*-RNAi line 13 still separated while *ATG4*-RNAi line 27 was grouped closely with WT, which is in agreement with the lower expression of *ATG4* in *ATG4*-RNAi line 13 and the dramatic changes of transcriptome and proteome results at green fruit stage (Figure 3 and 4).

We subsequently focused on metabolites which significantly changed in both lines 13 and 27 compared to WT. Among the primary metabolites of fruit, the first clear observation was the decreased trend of them in the *ATG4*-RNAi lines. For example, proline content was continually lower in the *ATG4*-RNAi lines during the three development stages, and tryptophan and phenylalanine were also decreased, especially at the green and mature green stages, respectively. Moreover, the content of arginine was just increased in the green stage and did not exhibit stable change during the following stages. Additionally, we did not detect many changes in sugars and organic acids levels between *ATG4*-RNAi lines and the control lines (Supplementary Data Set S1); however, a few observations were striking. First, *ATG4*-RNAi lines accumulated higher sucrose compared to WT at the red fruit stage. No significant difference was observed in glucose and fructose levels. Secondly, an increase of the sugar derivatives glucose-6-P and fructose-6-P had been detected in the *ATG4*-RNAi lines, especially the increase of glucose-6-P at the red fruit stage. As for sugars, we could not observe many significant changes in organic acids contents between *ATG4*-RNAi and control lines (Figure 2A, and Supplementary Data Set S1). One notable difference, however, was the low level of fumarate in *ATG4*-RNAi lines at the red fruit stage.

As the former results, green fruit stage was the key stage whose flavonoids, phenolics, and glycoalkaloids were highly accumulated in *ATG4*-RNAi lines while in the following stages, these metabolites exhibited an opposite change and decreased in *ATG4*-RNAi lines. Interestingly, the contents of the important secondary metabolite skeleton quinic acid and its derivatives, 3-trans-caffeoylquinic acid, were substantially greater in *ATG4*-RNAi green fruits than that of WT. Moreover, among the 44 dipeptides, we found that their levels were overall lower in *ATG4*-RNAi fruit in comparison to the control lines. In particular, Ala-Glu and Glu-Gln were significantly lower than that of WT fruit at the green fruit stage (Supplementary Data Set S1).

### Alterations in transcript levels and displayed by *ATG4*-RNAi tomato fruit

To obtain a genome-wide gene expression profile of the effect of *ATG4* on fruit, we checked differences in the transcriptome between the RNAi lines and WT fruit at green and red fruit stages. The FPKM (fragments per kilobase per million mapped fragments) values were calculated and genes with |log2 (fold change)| ≥ 1 and FDR (corrected-*p* value) < 0.05 were designated as differentially expressed genes (DEGs). At the green fruit stage, a total of 2952 (1279 up-regulated/1673 down-regulated) and 1269 (862 up-regulated/407 down-regulated) DEGs were identified in *ATG4*-RNAi-13 and *ATG4*-RNAi-27, respectively (Figure 3B and Supplementary Data Set S2). These results are in agreement with the significant difference in the plant’s early senescence phenotype (Figure 1A, B). Moreover, principal component analysis (PCA) based on the DEGs further confirmed the difference between *ATG4*-RNAi-13, *ATG4*-RNAi-27 and WT (Figure 3A). At the red fruit stage, *ATG4*-RNAi-27 fruits exhibited stable significant difference with WT with 1195 DEGs (1075 up-regulated/120 down-regulated) while in *ATG4*-RNAi-13 fruits just 169 DEGs (65 up-regulated/104 down-regulated) have been identified (Supplementary Data Set S3), and in the PCA analysis, *ATG4*-RNAi-13 samples were clustered with WT sample and separated from *ATG4*-RNAi-27 samples (Supplementary Figure S1).

We additionally analyzed the conserved DEGs in the two RNAi lines and found that 164 and 12 of them were up-regulated, 241 and 4 were down-regulated in the two lines at the green and red fruit stage respectively (Figure 3B and Supplementary Data Set S4). To analyse the functional categorization of DEGs, the conserved DEGs were further analyzed using AgriGO v2.0 analysis tools (http://bioinfo.cau.edu.cn/agriGO/) by Singular Enrichment Analysis (SEA) [25] and visualized by Mapman software [26] (Figure 3C, D). Among the four datasets (up/down conserved DEGs at green and red fruit stage), only the conserved downregulated DEG dataset at green fruit stage identified several significantly enriched pathways (FDR < 0.01). Among the GO terms included in the “biological process” category, the five most significantly enriched pathways were photosynthesis (FDR = 5.00x10^−15^), the reductive pentose-phosphate cycle (FDR = 6.70x10^−09^), photosynthesis (dark reaction) (FDR = 1.60x10^−08^), carbon fixation (FDR = 4.50x10^−08^) and photosynthesis (light reaction) (FDR = 3.20x10^−05^) (Figure 3D). Moreover, in the “cellular component” category, the top ten significantly enriched terms were related to photosynthetic membranes and chloroplasts (Supplementary Data Set S4). To further comfirm the transcriptome data, we have check the expression of representative genes involving in the lipid and photosynthesis process by qRT-PCR and the results also indicated that these genes such as *Lipase3*, *RBS3* and *LHCA6* were significantly down-regulated in the *ATG4*-RNAi lines (Figure 3B). These findings suggested that *ATG4* may operate as a key regulator of the photosynthetic organelle, chloroplast, and hence have an impact on fruit metabolism.

### Alterations in protein levels displayed by *ATG4*-RNAi tomato fruit

In order to check whether *ATG4* has similar effects on chloroplast-related genes at the protein level, total proteins of *ATG4-RNAi* lines and WT fruit at green and red fruit stages were analyzed by LC–MS/MS. In total, 2258 proteins were identified and at the green fruit stage, PCA indicated the significant difference between *ATG4*-RNAi-13, *ATG4*-RNAi-27 and WT (Figure 4A). In detail, 363 (160 up-regulated/203 down-regulated) and 283 (69 up-regulated/214 down-regulated) differentially abundant proteins (DAPs) (|log2 (FC)| > 1 and FDR < 0.05) were identified in *ATG4*-RNAi-13 and *ATG4*-RNAi-27 compared to WT fruit, respectively (Figure 4B, Supplementary Data Set S5). Unlike the transcriptome date, in red fruit stage, 259 DAPs (131 up-regulated/128 down-regulated) have been identified in *ATG4*-RNAi-13 fruits while just 71 DAPs (53 up-regulated/18 down-regulated) in *ATG4*-RNAi-27 compared to WT fruit (Figure 4B, Supplementary Data Set S6, Supplementary Figure S2).

Following the transcriptome analysis pipeline, only 45 conserved down-regulated DAPs at the green fruit stage identified several significantly enriched pathways (FDR ≤ 0.01) under the analysis of AgriGO v2.0 analysis tools and visualized by Mapman software [25] (Figure 4C, D). For the “biological process” category, photosynthesis is the most significantly enriched pathway, and in the “cellular component” category, the terms related to photosynthetic membrane and chloroplast are the most enriched terms, which was coincident with the transcriptome data (Figure 3D). Moreover, we also checked the conserved DEGs and DAPs of two RNAi lines, and just 6 genes and proteins exhibited significant down-regulation in the two *ATG4*-RNAi lines at the green fruit stage (Supplementary Data Set S7). Three of them, such as fructose-1,6-bisphosphatase, CRB (Chloroplast RNA binding) and NAD(P)-linked oxidoreductase, played important roles in photosynthesis chloroplast transcript and redox status of chloroplast (Supplementary Data Set S7). The transcriptome and proteomics data indicated that *ATG4* may affect the chloroplast function and then change the metabolism of the tomato fruit.

### Lipid profiling of *ATG4*-RNAi tomato fruits and correlation network analysis of metabolome, transcriptome and proteome

**Figure 5:**
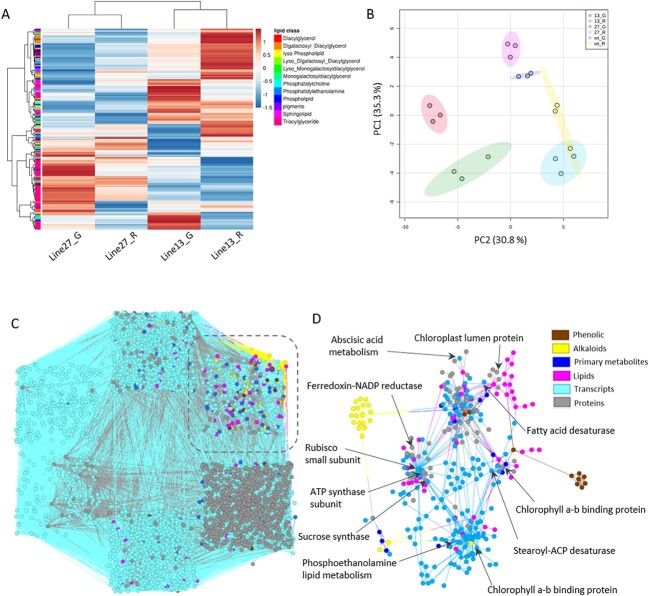
**Lipid profiling of ATG4-RNAi and correlation network analysis.** (A) heat map of lipid profiling of pericarp tomato fruit at green and red developmental stages. (B) a PCA of lipid profiling of WT and *ATG4*-RNAi plants from green and red fruits (G: green fruit and R: red ripe fruit. Log2 values of the relative lipids levels are presented as a heat map. The levels of ATG4-RNAi were normalized to the average of WT samples for each developmental stage. The lipid profiling was measured by LC–MS, lipids were divided into different lipid classes. (C) Correlation analysis of metabolome, transcriptome and proteome of fruit tissues of *ATG4*-RNAi and wild type plants. Genedata analyst was used to compute and generate graphical output of person correlation between metabolites, genes and proteins. Correlation network analysis, consists of 441 metabolites, 4684 transcripts and 953 proteins. (D) correlation network between 441 metabolites, 541 genes and 247 proteins involved in metabolism, chloroplast and photosynthesis.

To get more insight about the effect of *ATG4* downregulation on the fruit pericarp metabolism, we used ultra-high performance liquid chromatography–mass spectrometry (UPLC-MS) to determine the levels of 180 lipid compounds in green and red fruit tissues (Figure 5A, Supplementary Data Set S8). Principle component analysis (PCA) of the lipid profiling showed separation of the samples based on their developmental stages, in addition, separation was also observed between *ATG4*-RNAi lines and the WT in both developmental stages, with clear separation at the green fruit stages. This was more clear when compare the WT and *ATG4*-RNAi line 13. The changes in lipid profiling in the *ATG4*-RNAi lines compared to WT is shown in Figure 5B and statistical analysis showed significant changes in 91 and 12 in green stage and 57 and 12 lipid compounds in red ripe stage of *ATG4*-RNAi line 13 and line 27, respectively.

In order to look for correlations between metabolites, transcripts and proteins across *ATG4*-RNAi and wild type plants, we focused on fruit tissues and filtered our transcriptomic and proteomics data sets for genes showed significant difference between *ATG4*-RNAi and wild type plants. This resulting in 4684 transcripts and 953 proteins, in addition to 441 metabolites which used to calculate Pearson’s correlation between transcript, protein and metabolite profiles (Supplementary Data Set S9). Result revealed that many highly associations between and within transcripts and protein levels than metabolites and transcript and protein profiles (Figure 5C). To focus on the important interactions, we computed the correlation between all detected (441 metabolites), and 541 transcripts and 247 proteins that play a key role in metabolism, chloroplast and photosynthesis. Interestingly, we observed more and strong correlations between lipids (e.g triacylglyceride (TAG), monogalactosyldiacylglycerol (MGDG), and phosphatidylethanolamine (PE)) and genes involved in lipid metabolism, chlorophyll binding protein and chloroplast biosynthesis (Figure 5D). For examples, the rubisco small subunit (Solyc02g085950), ATP synthase subunit (Solyc06g066000), ferredoxin reductase (Solyc02g083810) involved in the Calvin cycle and photosynthesis were highly correlated and strongly connected to 13 lipid classes including TAGs, DGDGs and PEs. In addition, several transcripts and proteins involved in light harvesting complex of photosystem (e.g Solyc01g105030, and Solyc12g013710) were strongly correlated with genes involved in lipid metabolisms (e.g Solyc12g100230, Solyc08g082280, Solyc08g078400 and Solyc06g059720) and associated with various lipid classes.

### Characterization of metabolic fluxes in the *ATG4*-RNAi tomato fruit

In addition to measure the steady-state levels of transcripts, metabolites and proteins, we were interested in determining whether a deficiency in autophagy had major consequences on metabolic fluxes. For this purpose, we followed the metabolic fate of ^14^C- or ^13^C labelled glucose following incubation of tomato pericarp discs isolated from red ripe fruits. Evaluation of the data resulting from the ^14^C- experiment, which provided information concerning the major bulk fluxes in the cell, revealed few differences (Table 1), in that although the absolute fluxes to cell wall synthesis and protein biosynthesis were enhanced in line 27, no other fluxes were, and the relative redistribution of radiolabel to various metabolite classes were also unaltered. These data were somewhat reminiscent of those observed in etiolated seedlings of Arabidopsis, which also revealed relatively minor changes in the bulk fluxes [15]. However, by contrast to our observations here, the rate of protein synthesis alongside the rate of ^14^CO_2_ evolution was decreased on lack of autophagy in Arabidopsis. In parallel to this experiment, we additionally followed the metabolic fate of ^13^C labelled glucose using a GC–MS based protocol allowing a more detailed evaluation of changes in label redistribution amongst low molecular weight primary metabolites [27]. However, similar to ^14^C labelled glucose, the ^13^C labelled glucose shed minor changes between the WT and the *ATG4-*RNAi plants, with few significant metabolites enriched such as glutamate, putrescine, glycine and fructose (Supplementary Data Set S10).

**Table 1:** Metabolism of [U^14^C] Glucose in pericarp disks from WT and ATG4-RNAi plants

Variable	Control	Line 13	Line 27
Label incorporated (Bq/gFw)			
Total uptake	39 270 ± 12423.2	25801.4 ± 9356.9	56 264 ± 7948.2
Total metabolized	31 012 ± 9058.5	20 174 ± 6828.7	43 449 ± 6567.6
Redistribution of radiolabel (% of total metabolized)			
CO_2_ evolution	0.77 ± 0.25	5.45 ± 5.88	0.74 ± 0.12
Organic acids	45.72 ± 6.05	42.82 ± 2.71	50.37 ± 4.47
Amino acids	16.79 ± 3.81	19.73 ± 2.28	15.51 ± 1.84
Sucrose	11.68 ± 13.55	5.31 ± 0.95	6.98 ± 0.9
Starch	0.24 ± 0.23	0.49 ± 0.23	0.88 ± 0.38
Protein	0.42 ± 0.13	0.83 ± 0.34	0.39 ± 0.01
Cell wall	0.36 ± 0.1	0.62 ± 0.29	0.56 ± 0.13
Fructose	17 ± 11.07	8.63 ± 0.93	7.06 ± 3.03
Specific activity of hexose phosphates (Bq/nmol)	15.23 ± 3.9	17.48 ± 0.83	17.81 ± 5.14
Metabolic flux (nmol hexose equivalents/(gFw*h)			
Starch synthesis	1.01 ± 0.88	1.81 ± 0.77	5.12 ± 0.75
Sucrose synthesis	64.49 ± 91.05	21.06 ± 9.25	40.3 ± 15.24
Respiration	277.96 ± 128.24	223.39 ± 88.93	384.22 ± 142.28
Cell wall synthesis	1.53 ± 0.72	2.26 ± 0.84	**3.06 ± 0.49**
Protein synthesis	1.88 ± 1.08	3.39 ± 2.27	**2.21 ± 0.56**

## Disscussion

In leaves, the influence of autophagy on foliar metabolism has been widely studied [15, 18, 28–30]. This is especially true of Arabidopsis, where autophagy has been demonstrated to be important in nitrogen remobilisation [18, 30] as well as in the maintenance of energy metabolism under conditions of carbon starvation [15, 28]. However, recent studies in maize have allowed the generality of these studies to be determined [14, 29]. These studies revealed that autophagy played a key role for proteome remodelling and lipid turnover of leaves of young maize seedlings in nitrate replete and deficient conditions [14]. It furthermore played a prominent role in amino acid, nucleotide and carbohydrate metabolism during carbon starvation in the same system [29]. That said, as yet, studies of how autophagy impacts metabolism have not been carried out on the harvested tissue of a crop. Furthermore, to date, studies of the role of autophagy in tomato have been restricted to studying its role in the context of stress [31]. For this reason, we here performed a comprehensive characterization of the impact of downregulating autophagy by inhibiting *ATG4* gene expression and studying the effects on the whole plant phenotype, as well as on transcript, protein, and metabolite levels and metabolic fluxes under optimal growth conditions. In addition, in light of recent reports that autophagy is responsible for the accumulation of proteogenic dipeptides following heat stress in Arabidopsis [32], we also characterized the levels of these small molecules. By comparing these results with those previously reported for Arabidopsis and maize, we attempted here to define both the conserved and species−/ tissue- specific effects of inhibiting autophagy. Entirely consistently with previous studies, RNA interference of *ATG4* in tomato, resulted in early leaf senescence as well as a reduced yield (Figure 1D).

Given that most previous studies focused on foliar metabolism, it seems appropriate to firstly compare our results with those from other species. The primary metabolic changes observed in Arabidopsis mutants (*atg5*, *atg9,* and an *ATG18a* RNA interference line) 30 days after sowing were similarly minor as those we found here for young tomato leaves [30]. However, strikingly there was no overlap in the type of metabolites changing between young photosynthetically active tissues of both species (contrasting results from Figure 2A and Supplementary Data Set S1 with those for N replete conditions in [30]). Moreover, the Arabidopsis seedlings (60 days after sowing) exhibited changes in the levels of most metabolites [30], whilst changes in older tomato leaves remained moderate with changes in the level of phenylalanine being the only conserved change between the two studies (contrasting results from Figure 2A and Supplementary Data Set S1 with those for N replete conditions in [30]). Extensive metabolomics has also been carried out for *atg12* mutants of maize which exhibited considerable metabolic differences from the wild type in two-week-old maize seedlings. These studies demonstrated significant lipid remodelling as well as significant increase of amino acids, sugars, organic acids and nucleotides [29], thus as for Arabidopsis, it would appear that autophagy has a greater influence on primary leaf metabolism in these species than in tomato. Although this statement needs to be qualified in that distinct components of the autophagic machinery have been studied in the different species and the relative extent of inhibition of the process has yet to be ascertained. It’s worth noting that despite a considerably increased rate of leaf senescence, relatively small alterations in metabolite levels were found, which probably explains the conserved changes in the levels of phenylalanine which consistently alters under senescence, but moreover provides a strong suggestion that autophagy is considerably impaired given the well characterized role of autophagy within senescence [33].

**Figure 6:**
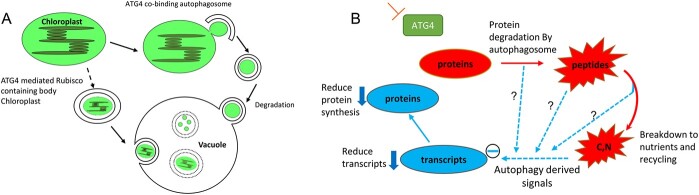
**A proposed model for the role of ATG4 in the chlorophagy process and the global autophagy process.** (A) Two routes were proposed for chlorophagy mediated by ATG4 on the basis of cell biological observations in ATG4 and ATG5 Arabidopsis knockout mutants (Yoshimoto et al. 2004). Our proteomics data are highly in support of ATG4 cholorphagy role being the one centered on chloroplast degradation. (B) Suppression of ATG4 results in the reduction of protein degradation by the autophagosome mediated vacuolanization of protein (in red). However, in addition to this canonical method of protein reduction the lack of correlation between transcript and protein pairs, suggests a second mediated operation (in blue) whereby protein synthesis is downregulated at the transcriptional level.

Despite the relatively mild changes in foliar metabolite levels, the strong transgenic lines harboured markedly less fruits and this led to a reduced total fruit biomass than found in the wild type. Since flowering and fruit set processes are greatly influenced by metabolite signals emanating from the source tissues [34] and tomato fruit growth is almost exclusively supported by imported assimilate rather than the operation of photosynthesis in the young fruit [35], it follows that the relatively minor changes in metabolite contents in the transgenics leaves may reflect the lower rate of partitioning between the source and sink tissues. Consistent with this hypothesis the changes in metabolites levels were considerably greater in the fruits and we therefore focused the majority of our efforts on characterizing this tissue. At the transcript level approximately 10% of expressed transcripts were found to be significantly different with a similar amount being up- and down-regulated at the green fruit stage but considerably fewer being altered at the red fruit stage (with a marked preference towards increased transcript levels also characterizing this developmental stage). Evaluating those transcripts which displayed conserved changes in both transgenic lines revealed that those classified by GO terms related to photosynthesis and the chloroplast were considerably enriched, suggesting that ATG4 may act as an important regulator of the photosynthetic organelle in fruit. As for the leaf metabolome, the changes in the fruit transcriptome are considerably different than those previously reported for the Arabidopsis rosette leaves which, under nitrogen replete conditions, was characterized by alterations in the pathways for glutathione, methionine, raffinose, galacturonate and anthocyanin [30]. Similarly, characterization of young maize seedlings under nutrient replete conditions identified similar pathways as being altered at the transcriptomic level but additionally noted an enrichment in alterations in the levels of transcripts associated with the cytosol, the endomembrane system, peroxisome and proteasome, but not the chloroplast. However, these differences could of course be due to differences between species, tissues, the function of the respective autophagy proteins that have been mutated, or the extent to which autophagy has been affected. It is of note that chloroplast breakdown has previously been linked to autophagy [36], either at the whole organelle level (“chloroplast autophagy”) [37], or as piecemeal degradation of selected chloroplast proteins (in RUBISCO containing bodies or ATI bodies) [38]. Moreover, the specific role of ATG4 was demonstrated at the cell biological level in the completely autophagy deficient *atg4a4b-1* mutant of Arabidopsis in which both downsizing due to the absence of RUBISCO containing bodies and lack of degradation of entire plastids [39]. However, it is important to note all these data were observed in leaves during developmental senescence or abiotic stress. To our knowledge, this is the first time autophagy in general, and ATG4 in particular, is implicated in chloroplast degradation during fruit development thus providing further support for previous models suggesting a considerable role of ATG4 in chloroplast autophagy (Figure 6A).

Despite the difference in the categories of transcripts/proteins tested between the species, it is notable that both for maize [14] and tomato there is a high correlation between the levels of proteins and transcripts belonging to the same class, although importantly not necessarily corresponding to the same gene. This is an important distinction since it suggests that at least some of these changes represent a co-ordinated response rather than merely being the consequence of a direct regulation at the level of transcription. We will return to this later, as alluded to above the proteins that change in the tomato ATG4 transgenics are largely associated with the chloroplast. In an earlier protein analysis of etiolated seedlings of *atg*5 and *atg*7 mutants we found an accumulation of proteins which increased respiration and reduced protein synthesis [15]. However, as recently reviewed by Wijerathna-Yapa and co-workers, a wide range of proteomic studies have been carried out [40] mainly in maize and Arabidopsis. In addition to the studies, we have already mentioned three further studies are worthy of discussion in this context. First, the study of *atg*10 Arabidopsis mutants upon infection of the fungus *Verticillum dahlia* revealed a specific enrichment of changes in the mitochondrial proteome. Secondly, a recent study utilized tandem mass tag labelling to identify autophagy cargo during cell fate switching in Arabidopsis [41]. Finally, immunoprecipitation in potato revealed that ATG8 isoforms display diverse interactomes [42], a fact that was extended in cross-kingdom studies of ER-phagy [43]. Consistently, with the predominance of plastid associated proteins in tomato the proteomic differentiation of the RNAi lines is far greater at the green than the red stage where its influence on tomato fruit metabolism and function is far lesser.

Analysis of fruit metabolite levels mirrored the conclusion made on the basis of the transcript and protein data that ATG4-dependent autophagy exerts its greatest influence at earlier stages of fruit development. As we already mentioned, this is in high accordance with the facts that its primary target – as assessed by the proteome – analysis would appear to be the chloroplast. Interestingly, the stress associated metabolite proline was decreased in both transgenic lines across all three fruit stages, as were tryptophan and phenylalanine, which are known to accumulate during the senescence process [44, 45], but moreover and were also documented to be considerably lower in etiolated seedlings of *atg5* and *atg7* Arabidopsis mutants [15]. Other commonalities between our study and that of the etiolated seedlings include the accumulation of sugars and sugar derivatives and in red fruits the ultimate decrease of secondary metabolites, however, it is important to note that these initially increased in the green fruit. Perhaps surprisingly given the recent finding that autophagy results in their accumulation following heat stress [32], we found relatively few significant changes in the levels of dipeptides and importantly none in the levels of Typ-Asp and Ser-Leu two peptides which have recently been reported to exert key roles in the regulation of glyceraldehyde 3-phosphate dehydrogenase and phosphoglycerate kinase of glycolysis, respectively [46, 47].

## Conclusions

The data we present here demonstrates an important role for ATG4-dependent autophagy within the tomato, suggesting that its absence leads to elevated leaf senescence most likely due to the change of other pathways of protein and organelle degradation such as the proteasome or chloroplast vesiculation pathways [28]. They also indicate that ATG4-dependent autophagy in tomato is quite distinct from that characterized for a range of autophagy mutants in Arabidopsis and maize – specifically it appears to target plastid related functions. Given its early senescence phenotype – as assessed by visual observation of the chlorophyll content -this appears to be true also in the leaf which was subjected to far less thorough characterization here. Despite affecting different proteins, strikingly our study revealed an interesting parallel to the recent multiomics studies of maize [14, 29], namely a co-ordinate change in classes of transcripts and proteins - albeit one that is largely not observed when comparing the direct transcript-protein pairs. This leads us to propose a more sophisticated model to explain the co-ordination one that suggest an alteration in protein degradation likely proceeds downregulation of transcripts (see Figure 6 B). However, given that autophagy has evolved to be highly embedded within cellular metabolism and function further studies likely involving inducible modulation of autophagy will likely be required both to establish the kinetics underlying the interplay between the various molecular entities of the cell. Such studies may ultimately identify the cellular signals that orchestrate the process to prevent energy wastage via unnecessary futile cycles of synthesis and degradation which would otherwise operate at many levels. Furthermore, given the major effect on the agronomic properties of tomato that this study uncovered a better understanding of the timing and maximal activities of autophagy during a normal life cycle may allow us to target the process as a means for crop improvement in future breeding strategies.

## Methods and materials

Tomato (*S. lycopersicum* L. cv Moneymaker) plants were provided by Meyer Beck (Berlin) and cultivared in the greenhouse.

### Construct design and plant transformation

220 bp of the coding sequence of tomato *ATG4* coding sequence (Solyc01g006230.2.1) were amplified by PCR (LP–CACCTTCAATTGATCCCTCCTTGG,RP– GGGGGCTACAGCATCGAA) and cloned into a TOPO vector. The sequence was then inserted using the Gateway system into the pK7GWIWG2(I) binary vector, used for the generation of a hairpin RNAi construct. The binary vector was transformed into competent *Agrobacterium tumefaciens* and subsequently transformed into M82 tomato callus. Transgenic plants were selected using Kanamycin and regenerated plants were grown in MS medium. Transgenic plants were verified by DNA extraction and amplification by PCR of the NPTII gene used for selection (LP- GATAGAAGGCGATGCGCTGCGAATC, RP- ATTCGGCTATGACTGGGCACAACAG).

### RNA sequencing and qRT-PCR anlysis

The RNA sequencing sequencing was carried out as the method of former research [48]. Briefly, total RNA (3 μg) was used to generate first-strand cDNA and the purified cDNA was subjected to Covaris shearing. End mending and A–tailing of sheared cDNA were done according to Illumina’s instructions. The libraries was sequenced by the Illumina Genome Analyzer IIx. FASTXToolkit 0.0.13 was used to filter the Illumina sequencing collection for quality. ITAG2.3 *S. lycopersicum* cDNA sequences were used to align quality-filtered libraries using Bowtie version 0.12.8 [49]. Total RNA and the first-strand cDNA synthesis was isolated and performed by TRIzol reagent and PrimeScript RT Reagent Kit with gDNA Eraser (Takara), respectively. qRT-PCR was performed on an ABI Prism® 7900 HT real-time PCR system (Applied Biosystems/Life Technologies, Darmstadt, Germany) in 384-well PCR plates. The qRT-PCR data were analyzed using the 2^−∆∆Ct^ analysis method according to the former research [50]. The information of primers is listed in the Supplementary Data Set S12.

### Metabolite and protein extraction

Pericarp samples were extracted using the former protocol [51]. For metabolite analysis 4–6 replicate samples were used. The protein data comes from three to four replicates.

### Proteomics analysis

Following the MTBE extraction, the protein pellets were resuspended in 50 μL of denaturation buffer. Alkylation was used to reduce cysteines, and enzymatic digestion was conducted with LysC/Trypsin Mix and desalted as reported before [52]. Peptides were resuspended in MS loading buffer and quantified using on a Q Exactive HF (Thermo Fisher Scientific) linked to an ACQUITY UPLC M-Class reverse-phase nano liquid chromatography system (Waters). A data-dependent acquisition strategy was used to run the MS.

### LC–MS metabolite analysis

The polar phases (400 μl) were vacuum-dried and resuspended in 120 μl of 80% MeOH. After sonicating for 10 minutes, samples were centrifuged at maximum speed for 15 minutes. For analysis, 100 μl of supernatant were examined using a UPLCLC-MS system, as previously reported [53]. Standard chemicals, MS/MS fragmentation, and metabolomics databases were used to identify and annotate metabolites. We accounted for a 10 ppm mass error and a 0.1 dynamic RT shift while utilizing our in-house reference compound library. For subsequent analysis, log2 normalized raw intensities were employed.

### GC–MS metabolite profiling

The MTBE-extracted polar phases (150 μl) were vacuum-dried and resuspended in 40 μL of methoxyamine hydrochloride (20 mg ml^−1^) in pyridine and incubated at 37°C for 120 min. After that, a 30 min treatment at 37°C with 70 μl of *N*-methyl-*N* (trimethylsilyl) trifluoroacetamide was carried out. A gas chromatograph was utilized in conjunction with a time-of-flight mass spectrometer in the GC–MS system. Peak area of each metabolite was standardized to the internal standard (ribitol) and the fresh weight.

### Lipid analysis

The MTBE phases (500 μL) was dried in speedVac concentrator and re-suspended in 250 μL acetonitrile: 2-propanol (7:3, vol/vol) solution. Two μL per sample was injected into Waters Acquity ultra-performance liquid chromatography system coupled with Fourier transform mass spectrometry (UPLC-FT-MS) in positive ionization mode. The analysis workflow included peak detection, retention time alignment and isotopic peaks from the MS data was performed as described in the former research [54].

### Correlation analysis

Metabolite data correlation was analyzed using the website MetaboAnalyst [55] and Expressionist Analyst 14.0.5 (Genedata, Basel, Switzerland) (https://www.genedata.com/products/expressionist). For the correlation analysis we used 441 detected metabolites, and 4684 and 953 differential transcripts and proteins, respectively. Person correlation was used, significant correlations (*P* ≤ 0.05) with >0.50 cut off were used to generate the network correlation. The detailed corlation anylsis present in Supplementary Data Set S9.

### [U-^14^C] glucose feeding experiments

Red ripe fruit pericarp were cut and incubated in 5 mL 10 mM MES-KOH, pH 6.5, containing 1.85 MBq/mmol [U-^**14**^C] glucose. After 2.5 hours of incubation on 100 rpm shaker, double-distilled water was used to wash multiple times of the material and frozen in liquid nitrogen. The evolved ^**14**^CO_2_ was collected in 0.5 mL of 10% (w/v) KOH. Extraction and fractionation were performed as previously described [56]. After ethanol extraction, the insoluble material was resuspended in water to count for starch.

### ^13^C glucose feeding experiments

Red ripe fruit pericarp were cut from red ripe fruit and incubated in 5 mL 10 mM MES-KOH, pH 6.5, containing 10 mM [^**13**^C]Glc. After 3 hours of incubation, double-distilled water was used to wash multiple times of the material and frozen in liquid nitrogen. Following that, samples were extracted in 100% methanol, and analyzed as former reported [27].

### Statistical Analysis

Student’s paired *t*-test was performed to assess whether the differences between two genotypes were statistically significant.

## Accession Numbers

The Sol Genomic Network locus number for the gene discussed in the article is Solyc01g006230 (ATG4), Solyc01g009870 (GDE1), Solyc02g063150 (RBS3), Solyc02g069620 (Rib5P Isomerase), Solyc02g088100 (EXP15), Solyc05g008290 (LPR1), Solyc06g060870 (Lipase 3), Solyc06g073260 (CSP41B), Solyc07g043570 (Aldo-keto reductase), Solyc08g007040 (GDCH), Solyc08g076220 (PRK), Solyc08g083360 (NDF4), Solyc09g011810 (FBPase), Solyc09g083150 (Ndh), Solyc12g009200 (LHCA6), Solyc12g010910 (Lipase), Solyc12g094640 (GAPB).

## Acknowledgments

S.A. and A.R.F. acknowledge funding of the PlantaSYST project by the European Union’s Horizon 2020 Research and Innovation Programme (SGA-CSA no. 664621 and no. 739582 under FPA no. 664620). F.Z acknowledges funding of National Natural Science Foundation of China (no. 32002102).

## Author contributions:

S.A., F.Z., J.V., T.Y., S.B., R.W., A.B., and T.A preformed the lab work. E.S., A.S. performed the proteomic analysis. S.A and F.Z performed the data analysis. A.R.F wrote the manuscript with input from all authors.

## Data availability statement.

The authors declare that all the data supporting the findings of this study are available within the paper and its supplemental information files.

## Conflicts of interest

The authors declare no conflicts of interest.

## Supplementary data

Supplementary data is available at *Horticulture Research * online.

## Supplementary Material

Web_Material_uhac129

## References

[1] Deter RL, Baudhuin P, De Duve C. Participation of lysosomes in cellular autophagy induced in rat liver by glucagon. J Cell Biol. 1967;35:C11–6. 6055998 10.1083/jcb.35.2.c11 PMC2107130

[2] Tsukada M, Ohsumi Y. Isolation and characterization of autophagy-defective mutants of Saccharomyces cerevisiae. FEBS Lett. 1993;333:169–74. 8224160 10.1016/0014-5793(93)80398-e

[3] Avin-Wittenberg T, Baluška F, Bozhkov PV et al. Autophagy-related approaches for improving nutrient use efficiency and crop yield protection. J Exp Bot. 2018;69:1335–53. 29474677 10.1093/jxb/ery069

[4] Marshall RS, Vierstra RD. Proteasome storage granules protect proteasomes from autophagic degradation upon carbon starvation. elife. 2018;7:e34532. 29624167 10.7554/eLife.34532 PMC5947986

[5] Klionsky DJ, Abdel-Aziz AK, Abdelfatah S et al. Guidelines for the use and interpretation of assays for monitoring autophagy (4th edition)(1). Autophagy. 2021;17:1–382. 33634751 10.1080/15548627.2020.1797280 PMC7996087

[6] Su W, Ma H, Liu C et al. Identification and characterization of two rice autophagy associated genes, OsAtg8 and OsAtg4. Mol Biol Rep. 2006;33:273–8. 17082902 10.1007/s11033-006-9011-0

[7] Pei D, Zhang W, Sun H et al. Identification of autophagy-related genes ATG4 and ATG8 from wheat (Triticum aestivum L.) and profiling of their expression patterns responding to biotic and abiotic stresses. Plant Cell Rep. 2014;33:1697–710. 24996626 10.1007/s00299-014-1648-x

[8] Zhou J, Wang J, Yu JQ, Chen Z. Role and regulation of autophagy in heat stress responses of tomato plants. Front Plant Sci. 2014;5:174. 24817875 10.3389/fpls.2014.00174 PMC4012191

[9] Dagdas YF, Pandey P, Tumtas Y et al. Host autophagy machinery is diverted to the pathogen interface to mediate focal defense responses against the Irish potato famine pathogen. elife. 2018;7:e37476. 29932422 10.7554/eLife.37476 PMC6029844

[10] Li F, Chung T, Pennington JG et al. Autophagic recycling plays a central role in maize nitrogen remobilization. Plant Cell. 2015;27:1389–408. 25944100 10.1105/tpc.15.00158 PMC4456646

[11] Minina EA, Moschou PN, Vetukuri RR et al. Transcriptional stimulation of rate-limiting components of the autophagic pathway improves plant fitness. J Exp Bot. 2018;69:1415–32. 29365132 10.1093/jxb/ery010 PMC6019011

[12] Bassham DC, Laporte M, Marty F et al. Autophagy in development and stress responses of plants. Autophagy. 2006;2:2–11. 16874030 10.4161/auto.2092

[13] Hofius D, Li L, Hafrén A, Coll NS. Autophagy as an emerging arena for plant-pathogen interactions. Curr Opin Plant Biol. 2017;38:117–23. 28545004 10.1016/j.pbi.2017.04.017

[14] McLoughlin F, Augustine RC, Marshall RS et al. Maize multi-omics reveal roles for autophagic recycling in proteome remodelling and lipid turnover. Nature plants. 2018;4:1056–70. 30478358 10.1038/s41477-018-0299-2

[15] Avin-Wittenberg T, Bajdzienko K, Wittenberg G et al. Global analysis of the role of autophagy in cellular metabolism and energy homeostasis in Arabidopsis seedlings under carbon starvation. Plant Cell. 2015;27:306–22. 25649436 10.1105/tpc.114.134205 PMC4456922

[16] Avin-Wittenberg T, Bajdzienko K, Wittenberg G et al. Global analysis of the role of autophagy in cellular metabolism and energy homeostasis in Arabidopsis seedlings under carbon starvation. Plant Cell. 2015;27:306–22. 25649436 10.1105/tpc.114.134205 PMC4456922

[17] Kurusu T, Koyano T, Hanamata S et al. OsATG7 is required for autophagy-dependent lipid metabolism in rice postmeiotic anther development. Autophagy. 2014;10:878–88. 24674921 10.4161/auto.28279 PMC5119067

[18] Guiboileau A, Yoshimoto K, Soulay F et al. Autophagy machinery controls nitrogen remobilization at the whole-plant level under both limiting and ample nitrate conditions in Arabidopsis. The New phytologist. 2012;194:732–40. 22404536 10.1111/j.1469-8137.2012.04084.x

[19] Di Berardino J, Marmagne A, Berger A et al. Autophagy controls resource allocation and protein storage accumulation in Arabidopsis seeds. J Exp Bot. 2018;69:1403–14. 29378007 10.1093/jxb/ery012 PMC6018931

[20] Carrari F, Baxter C, Usadel B et al. Integrated analysis of metabolite and transcript levels reveals the metabolic shifts that underlie tomato fruit development and highlight regulatory aspects of metabolic network behavior. Plant Physiol. 2006;142:1380–96. 17071647 10.1104/pp.106.088534 PMC1676044

[21] Li Y, Chen Y, Zhou L et al. MicroTom metabolic network: rewiring tomato metabolic regulatory network throughout the growth cycle. Mol Plant. 2020;13:1203–18. 32561360 10.1016/j.molp.2020.06.005

[22] Lira BS, Gramegna G, Trench BA et al. Manipulation of a senescence-associated gene improves fleshy fruit yield. Plant Physiol. 2017;175:77–91. 28710129 10.1104/pp.17.00452 PMC5580748

[23] Seo E, Woo J, Park E et al. Comparative analyses of ubiquitin-like ATG8 and cysteine protease ATG4 autophagy genes in the plant lineage and cross-kingdom processing of ATG8 by ATG4. Autophagy. 2016;12:2054–68. 27540766 10.1080/15548627.2016.1217373 PMC5103345

[24] Barros JAS, Cavalcanti JHF, Medeiros DB et al. Autophagy deficiency compromises alternative pathways of respiration following energy deprivation in Arabidopsis thaliana. Plant Physiol. 2017;175:62–76. 28710132 10.1104/pp.16.01576 PMC5580740

[25] Wang L, Zhang XL, Wang L et al. Regulation of ethylene-responsive SlWRKYs involved in color change during tomato fruit ripening. Sci Rep-Uk. 2017;7:16674. 10.1038/s41598-017-16851-y PMC5709409 29192231

[26] Usadel B, Poree F, Nagel A et al. A guide to using MapMan to visualize and compare omics data in plants: a case study in the crop species, maize. Plant Cell Environ. 2009;32:1211–29. 19389052 10.1111/j.1365-3040.2009.01978.x

[27] Roessner-Tunali U, Liu J, Leisse A et al. Kinetics of labelling of organic and amino acids in potato tubers by gas chromatography-mass spectrometry following incubation in (13)C labelled isotopes. The Plant journal : for cell and molecular biology. 2004;39:668–79. 15272882 10.1111/j.1365-313X.2004.02157.x

[28] Barros JAS, Cavalcanti JHF, Medeiros DB et al. Autophagy deficiency compromises alternative pathways of respiration following energy deprivation in Arabidopsis thaliana. Plant Physiol. 2017;175:62–76. 28710132 10.1104/pp.16.01576 PMC5580740

[29] McLoughlin F, Marshall RS, Ding X et al. Autophagy plays prominent roles in amino acid, nucleotide, and carbohydrate metabolism during fixed-carbon starvation in maize. Plant Cell. 2020;32:2699–724. 32616663 10.1105/tpc.20.00226 PMC7474275

[30] Masclaux-Daubresse C, Clément G, Anne P et al. Stitching together the multiple dimensions of autophagy using metabolomics and transcriptomics reveals impacts on metabolism, development, and plant responses to the environment in Arabidopsis. Plant Cell. 2014;26:1857–77. 24808053 10.1105/tpc.114.124677 PMC4079355

[31] Wang Y, Cai S, Yin L et al. Tomato HsfA1a plays a critical role in plant drought tolerance by activating ATG genes and inducing autophagy. Autophagy. 2015;11:2033–47. 26649940 10.1080/15548627.2015.1098798 PMC4824577

[32] Thirumalaikumar VP, Wagner M, Balazadeh S, Skirycz A. Autophagy is responsible for the accumulation of proteogenic dipeptides in response to heat stress in Arabidopsis thaliana. FEBS J. 2021;288:281–92. 32301545 10.1111/febs.15336

[33] Barros JAS, Magen S, Lapidot-Cohen T et al. Autophagy is required for lipid homeostasis during dark-induced senescence. Plant Physiol. 2021;185:1542–58. 33793926 10.1093/plphys/kiaa120 PMC8133563

[34] Ruan YL, Patrick JW, Bouzayen M et al. Molecular regulation of seed and fruit set. Trends Plant Sci. 2012;17:656–65. 22776090 10.1016/j.tplants.2012.06.005

[35] Lytovchenko A, Eickmeier I, Pons C et al. Tomato fruit photosynthesis is seemingly unimportant in primary metabolism and ripening but plays a considerable role in seed development. Plant Physiol. 2011;157:1650–63. 21972266 10.1104/pp.111.186874 PMC3327185

[36] Ishida H, Izumi M, Wada S, Makino A. Roles of autophagy in chloroplast recycling. Biochimica et Biophysica Acta (BBA)-Bioenergetics. 2014;1837:512–21. 24269172 10.1016/j.bbabio.2013.11.009

[37] Izumi M, Ishida H, Nakamura S, Hidema J. Entire photodamaged chloroplasts are transported to the central vacuole by autophagy. Plant Cell. 2017;29:377–94. 28123106 10.1105/tpc.16.00637 PMC5354188

[38] Fahnenstich H, Scarpeci TE, Valle EM et al. Generation of hydrogen peroxide in chloroplasts of Arabidopsis overexpressing Glycolate oxidase as an inducible system to study oxidative stress. Plant Physiol. 2008;148:719–29. 18685041 10.1104/pp.108.126789 PMC2556821

[39] Yoshimoto K, Hanaoka H, Sato S et al. Processing of ATG8s, ubiquitin-like proteins, and their deconjugation by ATG4s are essential for plant autophagy. Plant Cell. 2004;16:2967–83. 15494556 10.1105/tpc.104.025395 PMC527192

[40] Wijerathna-Yapa A, Stroeher E, Fenske R et al. Proteomics for autophagy receptor and cargo identification in plants. J Proteome Res. 2021;20:129–38. 33241938 10.1021/acs.jproteome.0c00609

[41] Rodriguez E, Chevalier J, Olsen J et al. Autophagy mediates temporary reprogramming and dedifferentiation in plant somatic cells. EMBO J. 2020;39:e103315. 31930531 10.15252/embj.2019103315 PMC7024839

[42] Zess EK, Jensen C, Cruz-Mireles N et al. N-terminal β-strand underpins biochemical specialization of an ATG8 isoform. PLoS Biol. 2019;17:e3000373. 31329577 10.1371/journal.pbio.3000373 PMC6675122

[43] Stephani M, Picchianti L, Gajic A et al. A cross-kingdom conserved ER-phagy receptor maintains endoplasmic reticulum homeostasis during stress. elife. 2020;9:e58396. 32851973 10.7554/eLife.58396 PMC7515635

[44] Araújo WL, Ishizaki K, Nunes-Nesi A et al. Identification of the 2-hydroxyglutarate and isovaleryl-CoA dehydrogenases as alternative electron donors linking lysine catabolism to the electron transport chain of Arabidopsis mitochondria. Plant Cell. 2010;22:1549–63. 20501910 10.1105/tpc.110.075630 PMC2899879

[45] Kamranfar I, Xue GP, Tohge T et al. Transcription factor RD26 is a key regulator of metabolic reprogramming during dark-induced senescence. The New phytologist. 2018;218:1543–57. 29659022 10.1111/nph.15127

[46] Luzarowski M, Vicente R, Kiselev A et al. Global mapping of protein-metabolite interactions in Saccharomyces cerevisiae reveals that Ser-Leu dipeptide regulates phosphoglycerate kinase activity. Commun Biol. 2021;4:181. 33568709 10.1038/s42003-021-01684-3 PMC7876005

[47] Moreno JC, Rojas BE, Vicente R et al. Tyr-asp inhibition of glyceraldehyde 3-phosphate dehydrogenase affects plant redox metabolism. EMBO J. 2021;40:e106800. 34156108 10.15252/embj.2020106800 PMC8327957

[48] Rallapalli G, Kemen EM, Robert-Seilaniantz A et al. EXPRSS: an Illumina based high-throughput expression-profiling method to reveal transcriptional dynamics. BMC Genomics. 2014;15:341. 24884414 10.1186/1471-2164-15-341 PMC4035070

[49] Langmead B, Trapnell C, Pop M, Salzberg SL. Ultrafast and memory-efficient alignment of short DNA sequences to the human genome. Genome Biol. 2009;10:R25. 19261174 10.1186/gb-2009-10-3-r25 PMC2690996

[50] Bustin SA, Benes V, Garson JA et al. The MIQE guidelines: minimum information for publication of quantitative real-time PCR experiments. Clin Chem. 2009;55:611–22. 19246619 10.1373/clinchem.2008.112797

[51] Giavalisco P, Li Y, Matthes A et al. Elemental formula annotation of polar and lipophilic metabolites using (13) C, (15) N and (34) S isotope labelling, in combination with high-resolution mass spectrometry. The Plant journal : for cell and molecular biology. 2011;68:364–76. 21699588 10.1111/j.1365-313X.2011.04682.x

[52] Sokolowska EM, Schlossarek D, Luzarowski M, Skirycz A. PROMIS: global analysis of PROtein-metabolite interactions. Current protocols in plant biology. 2019;4:e20101. 31750999 10.1002/cppb.20101

[53] Alseekh S, Tohge T, Wendenberg R et al. Identification and mode of inheritance of quantitative trait loci for secondary metabolite abundance in tomato. Plant Cell. 2015;27:485–512. 25770107 10.1105/tpc.114.132266 PMC4558650

[54] Zhu F, Alseekh S, Koper K et al. Genome-wide association of the metabolic shifts underpinning dark-induced senescence in Arabidopsis. Plant Cell. 2022;34:557–78. 34623442 10.1093/plcell/koab251 PMC8774053

[55] Pang Z, Chong J, Zhou G et al. MetaboAnalyst 5.0: narrowing the gap between raw spectra and functional insights. Nucleic Acids Res. 2021;49:W388–96. 34019663 10.1093/nar/gkab382 PMC8265181

[56] Szecowka M, Heise R, Tohge T et al. Metabolic fluxes in an illuminated Arabidopsis rosette. Plant Cell. 2013;25:694–714. 23444331 10.1105/tpc.112.106989 PMC3608787

