# Supplementary material for: Autophagy modulates the metabolism and growth of tomato fruit during development
Source: Hortic Res. 2022 Jun 13;9:uhac129. doi: 10.1093/hr/uhac129 (PMC9343920; doi:10.1093/hr/uhac129)

Supplemental Figure S1 Principal component analysis (PCA) of differential expressed genes between WT and *ATG4*-RNAi plants at red ripe fruit.

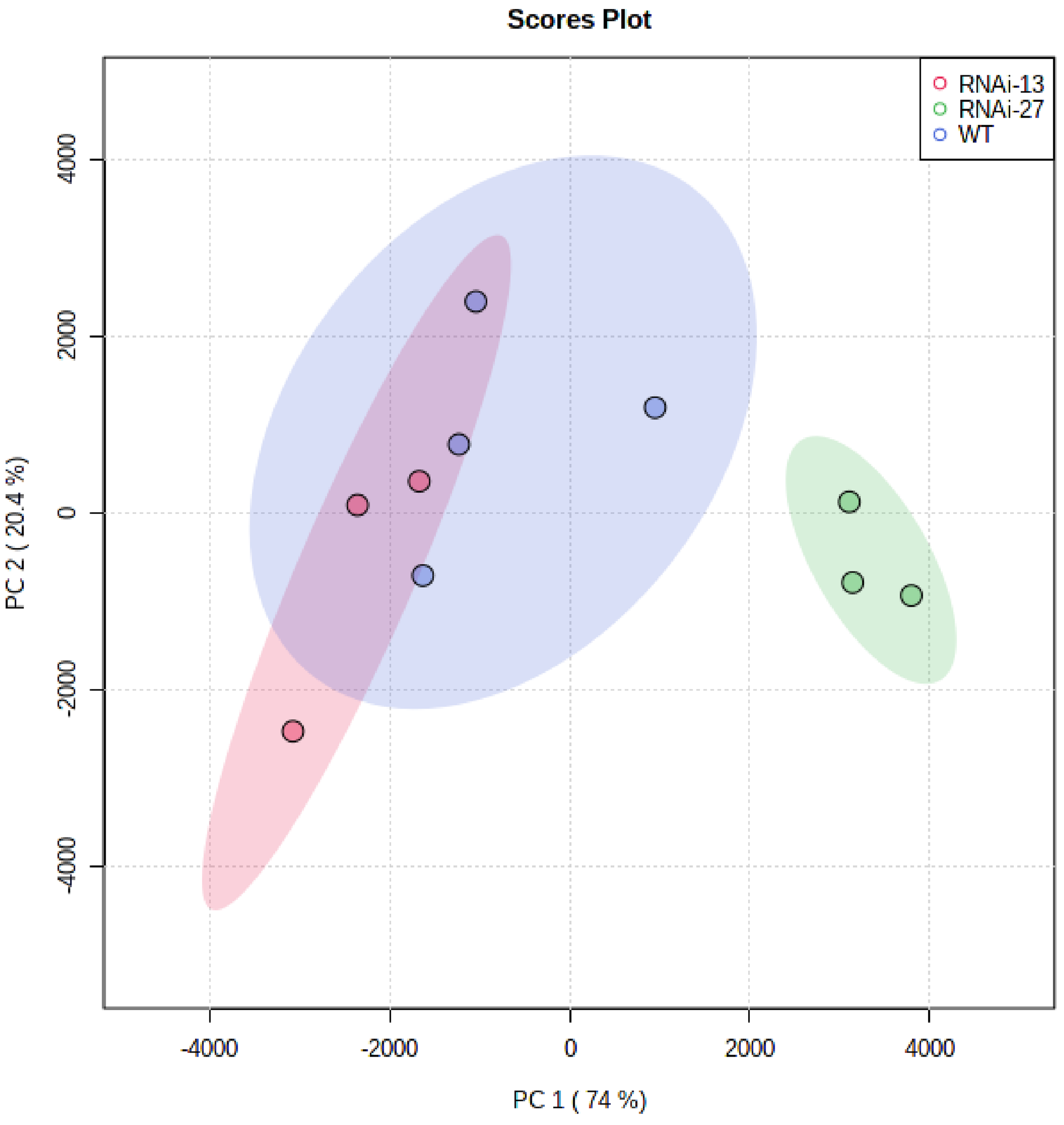

Supplemental Figure S2 Principal component analysis (PCA) of differential abundance proteins between WT and *ATG4*-RNAi plants at red ripe fruit.

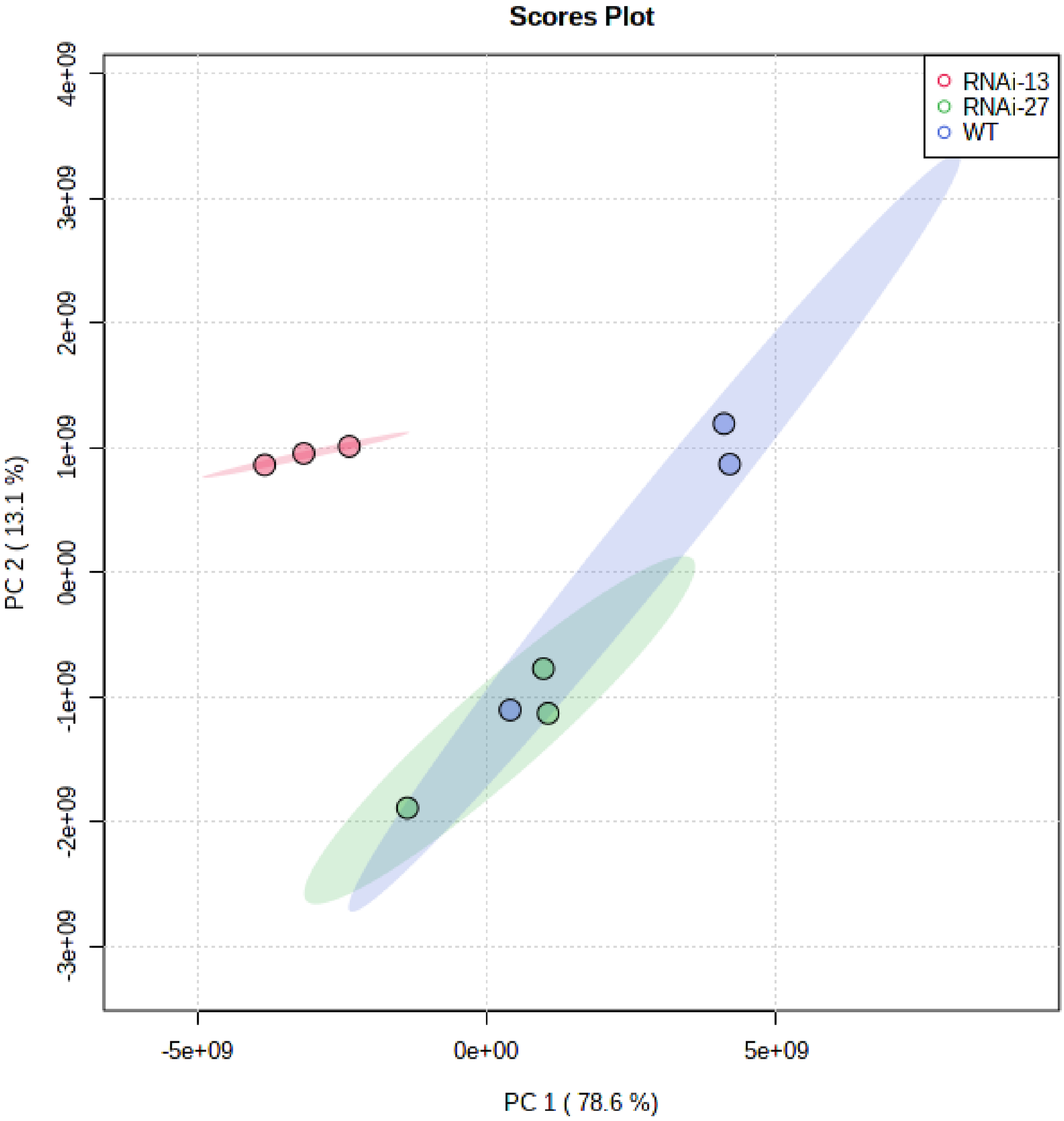

Supplement: Web_Material_uhac129 [file web_material_uhac129.zip › Supplementary Figures.pdf]
